# Molecular Characterization and Antifungal Susceptibility of *Aspergillus* spp. among Patients with Underlying Lung Diseases

**DOI:** 10.3390/tropicalmed7100274

**Published:** 2022-09-28

**Authors:** Ehssan Moglad, Samar Saeed, Humodi Saeed, Hind Ahmed, Kwathar Salih, Hisham Altayb, Wafa Elhag

**Affiliations:** 1 Department of Pharmaceutics, College of Pharmacy, Prince Sattam Bin Abdulaziz University, Al-Kharj P.O. Box 173, Saudi Arabia; 2 Department of Microbiology, Medicinal and Aromatic Plants and Traditional Medicine Research Institute (MAPTMRI), National Center for Research, Khartoum P.O. Box 2404, Sudan; 3 Department of Microbiology, Faculty of Medical Laboratory Sciences, Sudan University of Science and Technology, Khartoum P.O. Box 407, Sudan; 4 Department of Biochemistry, Faculty of Science, King Abdulaziz University, Building A 90, Jeddah P.O. Box. 80200, Saudi Arabia; 5 Centre for Artificial Intelligence in Precision Medicine, King Abdulaziz University, Jeddah P.O. Box. 80200, Saudi Arabia; 6 Department of Basic Medical Sciences, College of Medicine, University of Bisha, Bisha P.O. Box 1290, Saudi Arabia; 7 Department of Microbiology, Faculty of Medical Laboratory Sciences, Al-Neelain University, Khartoum P.O. Box 12702, Sudan

**Keywords:** pulmonary aspergillosis, antifungal, chronic pulmonary infection, PCR, Khartoum-Sudan

## Abstract

Background. Pulmonary aspergillosis is a lung infection caused by *Aspergillus* spp., which can cause severe illnesses in immunocompromised patients with underlying lung disease or who have asthma and inhale their spores. This study aimed to screen the antifungal susceptibility of *Aspergillus* spp. isolated from patients with underlying pulmonary infections and characterize the isolates using PCR and sequencing. Method. Three hundred and eighty-four sputum or bronchoalveolar lavage samples were collected and processed for the isolation and identification, and characterization of *Aspergillus* species and molecular amplification of the ITS1-5.8S-ITS2 region by the PCR and Sanger sequencing method. Antifungal susceptibility tests for itraconazole and voriconazole were performed using the E-test. Result. The overall results revealed that out of 384 patients, 32 (8.3%) were positive for fungal growth, including 28 (87.5%) *Aspergillus* spp. The highest resistance rate (100 and 44.4%) was obtained from itraconazole against *A. niger* and *A. fumigatus*. In contrast, voriconazole revealed the best activities against all tested fungi compared to itraconazole. All *A. flavus* were sensitive to voriconazole, while only 54.5% were sensitive to itraconazole. The MICs of E-test for *Aspergillus* spp were 1.6 ± 1.8 and 0.6 ± 0.93 for itraconazole and voriconazole, respectively. Conclusions. The prevalence of aspergillosis was high, with a significant association with underlying lung diseases. Voriconazole was the drug of choice for isolated fungi.

## 1. Introduction

An aspergillus pulmonary infection is a spectrum of various diseases in line with the host immunity. There are significant entities of invasive pulmonary aspergillosis and chronic pulmonary aspergillosis (CPA) [1,2,3,4], and three syndromes. The first syndrome is invasive aspergillosis (IA), which is now diagnosed to occur in sufferers with an essential infection without neutropenia and in those with moderate levels of immunosuppression, along with corticosteroid use within the setting of chronic obstructive pulmonary sickness (COPD). The second syndrome is CPA consisting of simple aspergilloma, which is now and again made complex by life-threatening hemoptysis and innovative damaging cavitary disorders requiring antifungal remedy [3,4]. The third syndrome is allergic bronchopulmonary aspergillosis (ABPA), an allergic reaction that is a response to Aspergillus mycelia that colonize the bronchi. Allergies and cystic fibrosis are commonplace ailments related to ABPA, with a reported prevalence of 1–2% in asthmatics, 7–14% in steroid-based asthmatics, and 2–15% in cystic fibrosis (CF) [5,6].

Historically, the occurrence of invasive aspergillosis has increased globally, consistent with the WHO, which recorded that worldwide around 1.2 million humans were estimated to have chronic pulmonary aspergillosis (CPA) in 2011 as a sequel to tuberculosis (TB). The highest cases were in the South-East Asian, Western Pacific, and African areas. Few facts are available on CPA as a publish-TB sequel and in structural lung sicknesses from growing countries [7,8,9].

Most chronic and allergic aspergillosis cases are misdiagnosed and treated as tuberculosis [7]. The population incidence of ABPA is not always, without a doubt, acknowledged, but the prevalence in asthma clinics is pronounced to be around 13% [10] and is the chief concern in CF patients [11]. Meanwhile, in humans with cystic fibrosis, the prevalence is 2% to 15% [12]. Allergic aspergillosis is clinically present with poorly managed asthma [13]. ABPA in allergies is an intense, lifestyle-affecting disease that probably impacts over 4.8 million people globally. ABPA predominantly results from the fungus *Aspergillus fumigatus* [14]. ABPA control consists of corticosteroids to govern the host immune reaction and antifungal drugs to lower the load of the organism. Itraconazole is the first-line agent for symptomatic ABPA sufferers based on randomized, controlled scientific trials. Voriconazole is an opportunity based totally on observational data [15].

Infections from Aspergillus species are highlighted as one of the predominant causative agents for illnesses. This likely results from a higher number of sufferers being at risk, such as transplant recipients, neutropenic people, allergic sufferers, and people dealing with corticosteroids or different immunosuppressive regimens [16]. *Aspergillus* spp. causes various immune-competence problems in addition to immune-compromised hosts, including allergic, colonizing, and invasive illnesses [17]. Itraconazole is used to prevent and remedy infections due to *A. fumigatus*. The knowledge of the pharmacodynamics of itraconazole towards wild-type and triazole-resistant strains gives a basis for progressive healing techniques to cure the disease [18].

PCR-based assays have been developed, and they can improve early diagnosis of aspergillosis with the advantages of high sensitivity, the ability to establish diagnosis at the species level, and the capacity to detect genes that confer antifungal resistance [19]. The target for genus-level detection of *Aspergillus* has included the 18S rRNA gene, mitochondrial DNA, the intergenic spacer region, and the internal transcribed spacer (ITS) regions. The latter is located between the 18S and 28S rRNA genes and offers distinct advantages over other molecular targets, including increased sensitivity due to the existence of approximately 100 copies per genome. The rRNA gene for 5.8S RNA separates the two ITS regions, and sequence variation of ITS regions has led to their use in phylogenetic studies of many different organisms [20]. The internal transcribed spacer rDNA region (ITS1-5.8S-ITS2) is the official DNA barcode for fungi because it is the most frequently sequenced marker in fungi and has primers that work universally [21]. This region can be used as the initial step of identification, including unknown fungal isolates to be categorized into appropriate genus, subgenus, and sections up to species level [22]. Although not translated into proteins, the ITS coding regions have a critical role in developing functional rRNA, with sequence variations among species showing promise as signature regions for molecular assays [23].

To the best of our knowledge, there is little awareness about fungal infections and pulmonary aspergillosis in Sudan. Moreover, there is a misdiagnosis of pulmonary aspergillosis with other pulmonary infections such as pulmonary tuberculosis and the effectiveness of itraconazole (the drug of choice) or other antifungals against *Aspergillus* species. Therefore, clinicians need to be familiar with clinical presentation, diagnostic methods, and pulmonary aspergillosis management. In Sudan, fungal infections in hospitals are much less common than other microbial infections, and susceptibility assessments for fungi are not routinely carried out. Consequently, this study aimed to show the incidence of aspergillosis, antifungal susceptibilities, and molecular characterization of *Aspergillus* spp. isolated from Sudanese patients with underlying pulmonary infections in the Khartoum state.

## 2. Materials and Methods

### 2.1. Study Population and Area

This study was a descriptive and cross-sectional study conducted in different centers and hospitals in Khartoum State at the mycology reference lab in the National Health Laboratory, Alshaab Teaching Hospital, Omdurman Chest Hospital, and the Police Hospital from February 2016 to February 2019. The study population was hospitalized or referred to patients with different age groups suffering from chronic respiratory diseases. All patients who experienced pulmonary symptoms, such as bronchiectasis, recurrent infections, chronic obstructive pulmonary diseases, cystic fibrosis, uncontrolled asthma, pulmonary tuberculosis, or a suspected case of fungus ball, were included in this study. Patients with acute pulmonary infections or with known bacterial pneumonia were excluded.

### 2.2. Data Collections and Sample Size

Data were collected through self and non-self-administrated questionnaires from patients. Some information was taken from the patient’s clinical reports. According to the below equation, 384 Bronchoalveolar lavages (BAL) and sputum specimens were collected.

(1) $$N=\frac{Z^{2}P\left(1-P\right)}{E^{2}}$$

P is the expected true proportion = 0.5, Z is the standard deviation corresponding to the desired confidence level (Z = 1.96 for 95% CI), and E is the desired precision (half desired CI width).

### 2.3. Specimens’ Collection and Processing

Early morning deep cough sputum specimens (341) were collected in sterile clean, dry containers for direct microscopy and culture, while bronchoalveolar lavages (BAL) (43) were collected in sterile containers by physicians. Then they were transported into an ice pack and processed as soon as possible [24].

### 2.4. Macroscopically and Microscopically Examinations

The color (yellow, green, or red), consistency (mucoid, mucopurulent, purulent, and bloody), and appearance of the specimens were observed [24].

#### 2.4.1. Direct Potassium Hydroxide (KOH)

All sputum and BAL samples were examined using potassium hydroxide preparations to provide descriptive morphological data of these pathogens to recognize fungal agents [24].

#### 2.4.2. Direct Gram Staining

One drop of normal saline was placed into a clear glass slide, and the purulent part of sputum was mixed with normal saline air-dried and by red heat fixed, stained using gram stain technique, and examined by the microscope using oil immersion. The smears were observed for the existence or absence of bacterial, yeast cells, and fungal elements.

### 2.5. Cultures of Specimens

In tow Sabroud Dextrose Agar, the purulent part of the sputum samples and BAL were inoculated and incubated at two different temperatures. One at 25 °C and the other at 37 °C for up to one week with daily examination for colonial morphology and indirect microscopy [24].

### 2.6. Identification of Isolates

The isolates were identified by macroscopic appearance (Surface topography, texture, and pigment). Characteristics of the colony include colony diameter after seven days, mycelia, exudates and reverse, colony texture, shape, and color of conidia [25].

#### Needle Mount with Lactophenol Cotton Blue (LPCB) Stain

Under the aseptic condition, one drop of lactophenol cotton blue stain was placed on a clean glass slide. A part of the colony was cut with a mycological needle and put in the slide, well mixed, covered, and examined under the microscope using (40×) to observe the conidial head of *Aspergillus* [24].

### 2.7. Antifungal Susceptibility Testing

#### 2.7.1. Preparation of Inoculum

The inoculum was prepared per the Clinical and Laboratory Standards Institute (CLSI) recommendation. The isolates were cultured on potato dextrose agar (PDA) slants at 35 °C for 2 to 7 days before testing for inducing sporulation. Then spores’ suspension was prepared by mixing the conidia into sterile saline containing two drops of tween 20 (0.05%). The turbidity of the suspension spectrophotometrically was adjusted to an optical density range from 0.09 to 0.11 at 530 nm (equivalent to106 spores/mL) [26].

#### 2.7.2. Seeding

Conidial suspensions swabbed into plates contain Roswell Park Memorial Institute (RPMI) 1640 medium encompassing L-glutamine and buffered to pH 7.0 with 0.165 M MOPS in three directions. When the agar surface was totally dry, E-test strips of voriconazole and itraconazole with concentrations ranging from 0.002 to 32 g/mL were implemented in the RPMI Dextrose agar [27].

#### 2.7.3. Reading and Interpretation of Results

Minimum inhibitory concentration (MIC) was reported using the E-test after 24 and 48 h incubation at 35 °C and defined as the drug concentration at which the elliptical inhibition zone’s border intersected the scale on the antifungal test strip. The result was interpreted according to Colosi et al. [28].

### 2.8. Molecular Identification of Aspergillus Isolates

#### 2.8.1. Culture Preparation and DNA Extraction Method

The isolated fungal species were cultured on a potato dextrose agar (PDA) medium and then incubated at 28 °C for 72 h. The DNA was extracted from the mycelium of pure culture colonies using the quick cetyltrimethylammonium bromide (CTAB) method [29]. DNA quality was checked via running 2–3 μL on an agarose gel (0.8%).

#### 2.8.2. PCR Method

A total of 5 μ from the test sample used for PCR assay, the total reaction volume was 25 μ ml containing PCR buffer (20 mMTris-HCl (pH 8.4), 50 mMKCl; 0.1 mM (each) dATP, dGTP, dCTP, and dTTP; 1.5 mM MgCl2; 0.3 mM (each) primer; and 1.5 U of PlatinumTaq high-fidelity DNA polymerase. Two oligonucleotide fungal primers were used for the amplification of the ITS region (ITS 1, 5′-TCC GTA GGT GAA CCT GCG G-3′; ITS 4, 5′-TCC TCC GCT TAT TGA TAT G-3′), which include the 18S (ITS 1) and the 28S (ITS 4) rRNA genes and the intervening 5.8S gene and the ITS 1 and ITS 2 noncoding regions [30]. Forty cycles of amplification were performed in a Heal Force T960 (China) thermocycler after the initial denaturation of DNA at 95 °C for 4.5 min. Each cycle consisted of a denaturation step at 95 °C for 30 s, an annealing step at 50 °C for 30 s, and an extension step at 72 °C for 1 min, with a final extension at 72 °C for 3 min following the last cycle. After amplification, the products were stored at 4 °C until used. The PCR products were analyzed on 2% agarose gel stained with ethidium bromide (10 ng/100 mL) and visualized under a UV transilluminator.

#### 2.8.3. DNA Sequencing

PCR products of 17 random isolates (only 17 due to financial constraints) were purified and shipped for the Sanger sequencing method using forward primers ITS1 and backward ITS4 by the BGI Company (Shenzhen, China).

### 2.9. Bioinformatics Analysis

#### 2.9.1. Sequences Similarity and Alignment

The BLAST sequence analysis tool was used to identify *Aspergillus* species through searching and comparing the sequences using nucleotide-nucleotide BLAST (BLASTn) with the default setting, except the sequences were not filtered for low complexity. Species were identified based on the reference database sequence’s highest similarity score (100%). Highly similar sequences were retrieved from NCBI and subjected to multiple sequence alignment using the BioEdit software [31]. All sequences were deposited to the GenBank with accession numbers found in the Table S1.

#### 2.9.2. Phylogenetic Tree

Phylogenetic tree of *Aspergillus* spp. amplified sequences were compared to reference strains from GenBank (NR_131276.1, NR_121481.1, NR_111041.1, AY373852.1 and EF661186.1). The MEGA program, version 7 [32], was used to generate the neighbor-joining method with bootstrap reconstructed 1000 times for tree reliability.

### 2.10. Data Analysis

Data were analyzed using the statistical package for social science software (SPSS IBM, version 0.20, Chicago, IL, USA). Frequencies, mean, and standard deviation were calculated. A Chi-square test was performed between qualitative variables, and an independent *T*-test was performed for Quantitative variables. A *p*-value ≤ 0.05 was considered significant for all statistical tests in this study.

## 3. Results

### 3.1. Socio-Demographic and Clinical Data

The distribution of three hundred eighty-four patients with underlying lung diseases enrolled in this study and clinical data are available in Table 1. Among the study population, the specimens’ frequency according to gender was 205 (53%) sputum specimens and 28 (7%) BAL specimens collected from males. Meanwhile, 136 (36%) sputum specimens and 15 (4%) BAL specimens were collected from females.

### 3.2. Microscopy and Isolation of Fungal Pathogens

Microscopically 11 (2.9%) sputum specimens were positive for fungal elements, 330 (85.9%) were negative, while all the BAL specimens were negative for fungi on direct microscopic examination. After primary isolation, out of 341 sputum specimens, 27 (7.9%) showed Aspergillus growth, 4 (1%) showed yeast growth, while 1/43 (2.3%) BAL specimens have grown as *Aspergillus*. Macroscopic and microscopic characteristics identified a total of 32 fungal isolates; 11 (34.4%) were *A. flavus*, 9 (28.1%) *A. fumigatus*, 7 (21.9%) *A. terreus*, 1 (3.13%) *A. niger* and 4 (12.5%) were *Candida* spp. The association between the underlying diseases and positive culture was significant, with a *p*-value of 0.003, as shown in Table 2.

Out of 28 Aspergilli isolates, 15 (46.9%) were isolated from patients with chronic pulmonary infections, 7 (29.1%) from asthmatic patients, 3(9.4%) from patients with cystic fibrosis, and 3 (9.4%) from pulmonary tuberculosis patients. The four (12.4%) Candida spp. were isolated from patients with malignancy and chronic pulmonary infections, two (6.2%) for each, with significant relationships between the underlying diseases and different fungal isolates (*p*-value = 0.015), as displayed in Table 3.

### 3.3. Antifungal Susceptibility Test

The highest resistance rate (100 and 44.4%) was obtained from itraconazole against *A. niger* and *A. fumigatus.* Voriconazole revealed the best activities against all tested fungi compared to itraconazole (Table 4). All *A. flavus* were sensitive to voriconazole, while only 54.5% were sensitive to itraconazole.

The MIC of itraconazole and voriconazole for *A. flavus* showed statistically significant differences between the two antifungals (*p*-value = 0.003). In contrast: *A. terreus* and *A. fumigatus* showed no statistically significant differences between the two drugs’ MIC (*p*-value = 0.08 and 0.6, respectively) (Table 4).

### 3.4. PCR Result for all Aspergillus Isolates

Isolated Aspergillus strains produced a PCR product of the ITS 1-5.8S-ITS 2 regions with sizes ranging from 565 to 613 bp.

### 3.5. Bioinformatics Analysis

**Figure 1:**
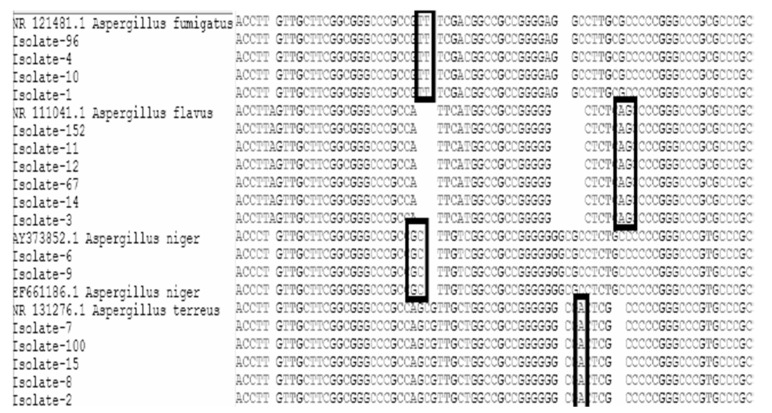
Nucleotide sequence alignment of *Aspergillus* isolates and reference strains in the gene bank; *A. flavus* (ATCC 16883), *A. fumigatus* (ATCC 1022), *A. niger* (ATCC 16888), *A. terreus* (ATCC 1012). The alignment consists of the 3′ end of the 18S ribosomal DNA (rDNA) gene (which contains the ITS 1 primer site), the entire ITS 1 region, the whole ITS2 region, and the 5′ end of the 28S rDNA gene (which contains the ITS 4 primer site). Black boxes indicated species-specific nucleotides.

**Figure 2:**
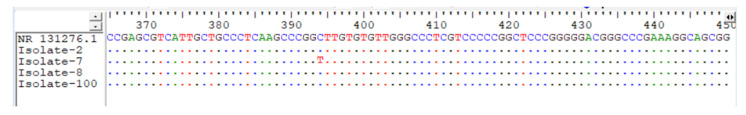
A difference of single nucleotide base between *A. terreus* clinical isolate and reference strain. The analysis was done using the BioEdit alignment editor v7.2.5.

**Figure 3:**
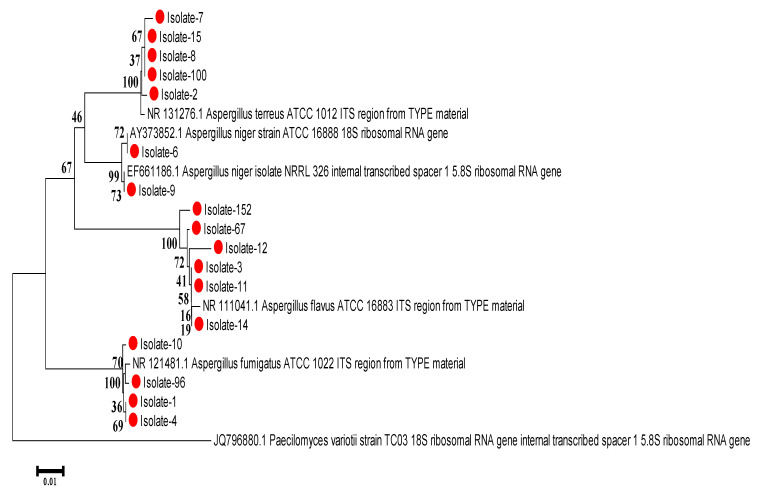
Phylogenetic tree based on interspaced gene sequences of 17 isolates (marked in red circles) from pulmonary aspergillosis patients. The phylogenetic tree analysis was constructed using the neighbor-joining method in MEGA 7. *Paecilomyces variotii* is used as the out-group to root the tree.

The PCR products of the ITS region of 17 isolates were sequenced and aligned with references in the NCBI database. The obtained sequences aligned with the Genbank reference strains (Figure 1). Comparisons between clinical isolates and reference sequences of the same Aspergillus species showed that most strains have 100% similarity to reference strains. One nucleotide base substitution (C/T) between the sequences of one clinical isolate and the reference strain was detected in isolate number 7 of *A. terreus* (Figure 2). The phylogenetic tree was constructed to compare the genetic distances and evolutionary lineage for 17 isolates with well-characterized reference isolates from Genbank (Figure 3). All generated sequences were submitted to Genbank, and the accession numbers are attached in the Table S1.

## 4. Discussion

Fungal infections have emerged as a global health care problem because of the massive use of wide-spectrum antibiotics, immunosuppressive agents, and growing debilitated sufferers. Aspergillus infection is the most common invasive fungal infection, including the respiratory tract [33]. Aspergillus-related lung diseases are traditionally dependent on the immunologic status of the host and the presence of underlying lung disease, patients with asthma or cystic fibrosis are affected by allergic bronchopulmonary aspergillosis (ABPA). Patients with abnormal airways are susceptible to aspergilloma, while invasive aspergillosis arises in severely immuno-compromised patients [34].

This study investigated the frequency of *Aspergillus* spp. and antifungal susceptibility among Sudanese patients with underlying pulmonary diseases. Chronic pulmonary infections were the most common underlying lung disease, while hemoptysis, lung abscess, and emphysema were the lowest. These findings agreed with a previous study conducted in Canada by AL-Alawy et al. (2005). They found that individuals with pre-existing structural lung disease, atopy, occupational exposure, or impaired immunity are susceptible [35].

In the direct microscopic examination, 11 (2.9%) sputum specimens were positive for fungal elements, 330 (85.9%) were negative, and all BAL specimens were negative for fungi. This result means the direct microscopic examination did not detect 21 out of 32 isolated fungi (65.6%), which were seen by culture. These are similar to that reported by Paugam et al. [36] in France (29%) and lower than those detected by Njunda et al. [37]. They found that the sensitivity of direct microscopy is 90%, while the Khodavaisy et al. [38] study showed that direct microscopy and culture remained negative in invasive pulmonary aspergillosis patients.

Interestingly, most of *Aspergillus* spp. was isolated from patients with pulmonary diseases and immunosuppression due to their higher risk of getting an infection. There was a significant association between the isolated species and pulmonary-related diseases (*p*-value = 0.003). These findings are inconsistent with several studies; Kosmidis and Denning (2015) reported that invasive aspergillosis (IA) predominantly occurred in patients with weaknesses in the immune system and CPA present with underlying lung disease but with no or only obvious generalized immune compromise [39]. Another study done by Ader and his colleagues (2006) reviewed that chronic obstructive pulmonary disease (COPD) is a third significant influencing factor for acute invasive pulmonary aspergillosis [40].

A higher frequency of isolates was found in chronic pulmonary patients, followed by asthmatic patients, and equally in cystic fibrosis and pulmonary tuberculosis. These results agreed with the study by Khlaif Imran (2015), who found that *Aspergillus* spp. was the major fungal pathogen identified in patients with pulmonary diseases, particularly those with COPD and pulmonary TB [41].

In this study, *A. flavus* was the most predominant isolate in chronic pulmonary infection and pulmonary tuberculosis. In contrast, Tashiro and his colleagues (2011) reported that *A. niger* was the most frequent isolate, followed by *A. fumigatus*, *A. versicolor*, and *A. terreus* from patients with ABPA [42].

Moreover, this study determined the excellent effectiveness of voriconazole against *A. flavus* and *A. fumigatus* and suggested that voriconazole may be the treatment of choice in pulmonary aspergillosis caused by these fungi; similar results were reported [43,44]. Unlike other studies, voriconazole and itraconazole had the lowest MICs against *Aspergillus* spp. compared to other fungi included in his study [45].

In this study, fragments of the ITS1-5.8S-ITS2 were amplified by the use of the primers ITS1 and ITS4 universal primers for the identification of *Aspergillus* species; 22/28 (78.6%) *Aspergillus* species were confirmed by using the primers ITS1 and ITS4, 6 (21.4%) isolates were negative. The negative results may be attributed to the isolated species not being genetically the same so that it can be detected by ITS1 and ITS4, or a mutation occurred with the isolated species and changed the nucleotide sequence and was not associated with the mentioned primer [46]. Many similar studies were done to detect and identify fungi using the internal transcribed spacer (ITS) and were published by several scientists [47,48,49].

Based on this study, we recommended that chronic pulmonary infections, particularly relapse and treatment failures, be subjected to fungal investigation to reduce the disease burden and improve clinical management.

## 5. Conclusions

This study concluded a significant correlation between frequencies of Aspergillus spp. in patients with chronic pulmonary underlying diseases. *A. flavus* was the most dominant *Aspergillus* spp. isolate. Furthermore, voriconazole antifungal was more active than itraconazole, especially against *A. flavus.*

## Figures and Tables

**Table 1:** Socio-demographic and clinical data of patients who participated in the study.

Variables	Total *n* (%)	*Aspergillus* spp.	
		Negative	Positive
		(*n* = 356)	(*n* = 28)
Sex:			
Male	233 (61.0)	23	210
Female	151 (39.0)	5	146
Specimens:			
Sputum	341 (89)	27	314
Bronchoalveolar Lavage	43 (11)	1	42
Underlying lung diseases:			
Chronic pulmonary infection	219 (57.0)	15	204
Asthma	77 (20.1)	7	70
Cystic fibrosis	34 (8.9)	3	31
Pulmonary tuberculosis	29 (7.6)	3	26
Pleural effusions	12 (3.1)	0	12
Malignancy	9 (2.3)	0	9
Emphysema, hemoptysis and lung abscess	4 (1.0)	0	4
Age group:			
<10	2 (0.5)	0	2
11–49	258 (67.2)	22	236
≥50	124 (32.2)	6	118

**Table 2:** Correlation between positive culture results and the underlying lung diseases.

Underlying Lung Diseases	Aspergillosis	Candidiasis	Total
**CPI (** ***n*** **= 219)**	15 (46.9%)	2 (6.2%)	17 (53.1%)
**Asthma (** ***n*** **= 77)**	7 (21.9%)	0 (0.0%)	7 (21.9%)
**CF (** ***n*** **= 34)**	3 (9.4%)	0 (0.0%)	3 (9.4%)
**PTB (** ***n*** **= 29)**	3 (9.4%)	0 (0.0%)	3 (9.4%)
**Malignancy + pleural effusions (** ***n*** **= 21)**	0 (0.0%)	2 (6.2%)	2 (6.2%)
**Total**	28 (87.5%)	4 (12.5%)	32 (100.0%)

A significant association between positive culture and the underlying lung diseases *p*-value = 0.003. Key: CPI: chronic pulmonary infection, CF: cystic fibrosis, PTB: pulmonary tuberculosis.

**Table 3:** Correlation between the underlying diseases and isolated organisms.

Disease	Isolates					
	*A. fumigatus*	*A. flavus*	*A. terreus*	*A. niger*	*Candida* spp.	Total
**CPI (** ***n*** **= 219)**	6 (18.8%)	6 (18.8%)	3 (9.4%)	0 (0.0%)	2 (6.2%)	17 (53.1%)
**Asthma (** ***n*** **= 77)**	3 (9.4%)	2 (6.2%)	2 (6.2%)	0 (0.0%)	0 (0.0%)	7 (21.9%)
**CF (** ***n*** **= 34)**	0 (0.0%)	0 (0.0%)	2 (6.2%)	1 (3.1%)	0 (0.0%)	3 (9.4%)
**PTB (** ***n*** **= 29)**	0 (0.0%)	3 (9.4)	0 (0.0%)	0 (0.0%)	0 (0.0%)	3 (9.4%)
**Malignancy + pleural effusions (** ***n*** **= 21)**	0 (0.0%)	0 (0.0%)	0 (0.0%)	0 (0.0%)	2 (6.2%)	2 (6.2%)
**Total**	9 (28.1%)	11 (34.4%)	7 (21.9%)	1 (3.1%)	4 (12.5%)	32 (100.0%)

A significant association between underlying lung diseases and isolated organism *p*-value = 0.015. Key: CPI: chronic pulmonary infection, CF: cystic fibrosis, PTB: pulmonary.

**Table 4:** Antifungal susceptibility patterns and MIC of *Aspergillus* spp. using the E test.

Isolates (n)	Itraconazole	Voriconazole	*p*-Value
*A. fumigatus* (9)	R: 4 (44.4%)	R: 1 (11.1%)	0.08
	I: 2 (22.3%)	I: 1 (11.1%)	
	S: 3 (33.3%)	S: 7 (77.8%)	
MIC g/mL	0.047–6	0.008–4	
*A. flavus* (11)	R: 1 (9.1%)	R: 0	0.003 *
	I: 4 (36.4%)	I: 0	
	S: 6 (54.5%)	S: 11 (100%)	
MIC g/mL	0.125–4	0.064–1.5	
*A. terreus* (7)	R: 1 (14.3%)	R: 1 (8.4%)	0.06
	I: 1 (14.3%)	I: 1 (8.3%)	
	S: 5 (71.4%)	S: 5 (83.3%)	
MIC g/mL	0.125–2.5	0.125–3	
*A. niger* (1)	R: 1 (100%)	I: 1 (100%)	
MIC g/mL	8	1.5	

Key: n = total number of isolated fungi, R: resistant, I: intermediate, S: sensitive. * is statistically significant; *p*-value = 0.003

## Data Availability

All data generated or analyzed during this study and accession numbers of GenBank submitted sequences were included in this published article and its Supplementary Information Files.

## Supplementary Materials

The following supporting information can be downloaded at: https://www.mdpi.com/article/10.3390/tropicalmed7100274/s1, Table S1: Gene Bank Accessions Number of isolated *Aspergillus* spp.
