# Supplementary material for: Molecular Characterization and Antifungal Susceptibility of Aspergillus spp. among Patients with Underlying Lung Diseases
Source: Trop Med Infect Dis. 2022 Sep 28;7(10):274. doi: 10.3390/tropicalmed7100274 (PMC9612272; doi:10.3390/tropicalmed7100274)
Supplement: Supplementary file 1 [file tropicalmed-07-00274-s001.zip › tropicalmed-1931270-supplementary.pdf]

Tropical Medicine and Infectious Disease

MDPI

Supplementary Materials for Molecular Characterization and Antifungal Susceptibility of *Aspergillus* spp. among Patients with Underlying Lung Diseases

Table S1. Gene Bank Accessions Number of isolated *Aspergillus* spp.

	Label	Accessions Number
SUB9090415	Isolate-1	MW604197
SUB9090415	Isolate-2	MW604198
SUB9090415	Isolate-3	MW604199
SUB9090415	Isolate-4	MW604200
SUB9090415	Isolate-6	MW604201
SUB9090415	Isolate-7	MW604202
SUB9090415	Isolate-8	MW604203
SUB9090415	Isolate-9	MW604204
SUB9090415	Isolate-10	MW604205
SUB9090415	Isolate-11	MW604206
SUB9090415	Isolate-12	MW604207
SUB9090415	Isolate-14	MW604208
SUB9090415	Isolate-15	MW604209
SUB9090415	Isolate-67	MW604210
SUB9090415	Isolate-96	MW604211
SUB9090415	Isolate-100	MW604212
SUB9090415	Isolate-152	MW604213
